# Assessing the Public Health Implications of Virulent and Antibiotic-Resistant Bacteria in Côte d'Ivoire's Ready-to-Eat Salads

**DOI:** 10.1155/2024/3264533

**Published:** 2024-08-06

**Authors:** N'goran Parfait N'zi, Valérie Carole Gbonon, Kipré Bertin Guédé, Sidjè Arlette Afran, Djédoux Maxime Angaman

**Affiliations:** ^1^ Department of Biochemistry-Microbiology Jean Lorougnon Guede University, Daloa, Côte d'Ivoire; ^2^ Department of Bacteriology-Virology National Reference Center for Antibiotics Pasteur Institute of Côte d'Ivoire, Daloa, Côte d'Ivoire

## Abstract

In Côte d'Ivoire, the popularity of ready-to-eat salads has grown substantially. Despite their convenience, these products often face criticism for their microbiological safety. This research was conducted to assess the virulence and antibiotic resistance profiles of *Escherichia coli* (*E. coli*), *Salmonella spp*., and *Staphylococcus aureus* (*S. aureus*) isolated from salads available in hypermarkets across Abidjan. The study utilized a combination of microbiological and molecular biology techniques. Results indicated that *E. coli* isolates harbored virulence genes such as *stx2* (50%) and *ST* (62.50%), though genes *stx1* and *LT* were absent in the samples tested. In *S. aureus*, virulence genes detected included *sea* (55.55%), *sec* (11.110%), and *sed* (44.44%). The antibiotic resistance assessment revealed high resistance in *E. coli* to *β*-lactam antibiotics, with all isolates resistant to cefuroxime (100%) and the majority to ampicillin and cefoxitin (87.5%). Most *Salmonella spp.* isolates were sensitive to the antibiotics tested, except for cefoxitin and ampicillin, showing resistance rates of 42.85% and 57.15%, respectively. *Staphylococcus aureus* demonstrated considerable resistance, particularly to cefoxitin (44.44%), benzylpenicillin (100%), and ampicillin (55.55%). In addition, resistance to aminoglycosides (55.55% to both kanamycin and gentamicin) and macrolides (66.66% to erythromycin and 55.55% to clindamycin) was noted. Resistance to various fluoroquinolones ranged between 33.33% and 55.55%. The presence of resistance genes such as *blaTEM* (10.52%), *qnrA* (2.26%), *qnrB* (5.26%), *qnrS* (5.26%), and *mecA* (13.15%) in *E. coli* and *S. aureus* underscores the challenge of multidrug resistance, exhibiting phenotypes such as ESBL (50%), Meti-R (55.55%), KTG (44.44%), MLSB (44.44%), and FQ-R (25%). These results carry significant epidemiological and public health implications, highlighting the urgent need for improved safety regulations and practices regarding ready-to-eat salads in urban food markets.

## 1. Introduction

Ready-to-eat fruit and vegetables, also known as 4th-range products, appeared in European supermarkets from 1980 onwards [1]. According to WHO, FAO, and the World Cancer Research Fund, the consumption of 400–600 g of fruit and vegetables a day is recommended [2, 3]. A diet rich in fruits and vegetables is likely to reduce the risk of cardiovascular disease and protect against certain types of cancer [4]. However, several cases of food poisoning associated with the consumption of these products have been observed around the world. For example, in 2012, a foodborne outbreak was reported in a college in China due to the consumption of salad ingredients [5]. In May 2014, an outbreak was observed in military and civilian populations associated with the consumption of ready-to-eat mixed salad in Norway [6]. In April 2019, a cross-border outbreak in Sweden including 37 cases and 20 cases in Denmark following consumption of imported fresh spinach was reported [7]. Based on currently available statistics, bacterial species in this case, *Escherichia coli (E. coli), Salmonella spp.*, and *Staphylococcus aureus (S. aureus)*, are among the important pathogens associated with fourth-range foods [8]. In Africa, the average prevalence of *E. coli* in ready-to-eat foods is 31.6%. The prevalence of *Salmonella spp.* is estimated at 21.7% and that of *S. aureus* at 25.1% [9]. Today, in developing countries such as Côte d'Ivoire, the consumption of ready-to-eat salads has become a cause for concern. Indeed, the desire to eat well is now a general trend [10]. However, high prevalences of *E. coli* (16%), *Salmonella spp.* (18%), and *S. aureus* (24%) have been determined in ready-to-eat salads sold in the city's supermarkets [11]. Thus, this work was carried out to determine the levels of virulence and antibiotic resistance of *E. coli*, *Salmonella spp.*, and *S. aureus* isolates isolated from ready-to-eat salads sold in Abidjan supermarkets. This work aims to prevent the dissemination of virulence and resistance genetic material of these pathogens through the consumption of these foods in Côte d'Ivoire.

## 2. Materials and Methods

### 2.1. Study Area

This study examines industrially produced ready-to-eat salads available in large supermarkets within Abidjan, Côte d'Ivoire. Abidjan is located in the southern part of the country, along the Gulf of Guinea, and covers an area of 2119 km^2^, representing 0.6% of the national territory, with a population density of 1475 inhabitants per km^2^. Sample analyses were conducted in the laboratory at Université Jean Lorougnon Guédé in Daloa, the capital of the Haut-Sassandra region. Positioned centrally in the western part of Côte d'Ivoire, Daloa is situated at a latitude of 6°52′38″ north and a longitude of 6°27′00″ west, approximately 141 km from Yamoussoukro, the political capital and 383 km from Abidjan, the economic hub. Further studies on antibiotic resistance were carried out at the Bacteriology-Virology Department of the Institut Pasteur de Côte d'Ivoire, within the Antibiotics, Natural Substances, and Surveillance of Microorganisms and Antibiotics Unit (ASSURMI), located in Cocody.

### 2.2. Biological Material

This experimental study analyzed bacterial isolates from 38 samples of ready-to-eat salads collected from hypermarkets in Abidjan, Côte d'Ivoire. These different samples come from 5 supermarkets located in the communes of Cocody (3) and Marcory (2). The research focused on three bacterial species: *Escherichia coli* (8 isolates), *Salmonella spp*. (7 isolates), and *Staphylococcus aureus* (9 isolates). These isolates, previously characterized using conventional microbiological techniques, were sourced from a diverse range of salad types as detailed in Table 1.

### 2.3. Searching for Virulence Genes

#### 2.3.1. Preparation of Genetic Material (DNA)

DNA extraction was performed using the CTAB method, as outlined in reference [12]. The process began by centrifuging 1.5 mL of the bacterial preculture in LB medium at 16,000 rpm for 5 minutes to pellet the cells. After discarding the supernatant, the pellet was resuspended in 1.5 mL of CTAB1 extraction buffer (20 g/L CTAB, 1.4 mol/L NaCl, 0.1 mol/L Tris, 0.02 mol/L Na-EDTA, and a pH of 8.0) and 5 *μ*L of RNAse (20 mg/mL). This mixture was then incubated at 60°C for 30 minutes, with intermittent shaking to resuspend the material. Proteinase K (10 *μ*L of 20 mg/mL) was added halfway through the incubation.

Following another round of centrifugation, 900 *μ*L of the supernatant was extracted and mixed with an equal volume of chloroform. After vortexing and subsequent centrifugation at 15, 000*g* for 15 minutes, 650 *μ*L of the clear supernatant was mixed with 1.3 mL of CTAB2 precipitation buffer and left to stand at room temperature for 60 minutes. Postcentrifugation, the supernatant was discarded, and the DNA pellet was washed with a NaCl solution (700 *μ*L of CTAB3) followed by chloroform. The aqueous phase was then mixed with twice its volume of cold isopropanol and allowed to precipitate at room temperature for 20 minutes.

The DNA was then pelleted by centrifugation, washed with 70% ethanol, dried in an oven at 55°C for 30 minutes, and finally resuspended in 30 *μ*L of TE buffer. The extracted DNA was stored at −20°C for further analysis.

#### 2.3.2. Amplification of Desired Genes

For *E. coli*, the target genes included those encoding Shiga toxins 1 and 2 (*stx1* and *stx2*), which were amplified using multiplex PCR. The amplification also targeted genes for heat-labile (*LT*) and heat-stable (*ST*) toxins. The PCR mix, based on the protocol described in [13], consisted of 20 *μ*L total volume: 10 *μ*L of 2X Phusion Master Mix, 2 *μ*L of primers (both sense and antisense, detailed in Table 2), 2 *μ*L of extracted DNA, and 6 *μ*L of sterilized nuclease-free water. The PCR conditions included an initial denaturation at 94°C for 5 minutes, followed by 30 cycles of amplification (details in Table 3). The PCR products, stained with 1 *μ*L of 6X loading buffer, were analyzed on a 1.2% agarose gel.

For *S. aureus*, the study focused on characterizing five stereotyped enterotoxins (*sea*, *seb*, *sec*, *sed*, and *see*), which are heat-stable proteins linked to food poisoning. The virulence genes were amplified in two multiplex sets: one for *sea* and *seb* and another for *sec*, *sed,* and *see*, using primers listed in Table 3. Amplifications were conducted in a mini thermal cycler (miniPCR bio^TM^), following the protocol in [15]. The resultant PCR products were subjected to electrophoresis on a 2% agarose gel.

### 2.4. Study of Antibiotic Resistance

#### 2.4.1. Determination of Resistance Profile

This component of the study involved conducting antibiograms using the agar diffusion method (Mueller Hinton, MH) as outlined by the antibiogram committee of the Société Française de Microbiologie [16]. Initially, a 24-hour culture of each isolate was prepared. Subsequently, a bacterial suspension was made in a 2 mL solution of 0.85% NaCl to match the 0.5 McFarland standard, equivalent to approximately 1 to 2 × 10^8^ CFU/mL. Inoculation was performed on the surface of MH agar plates, followed by the application of antibiotic discs, considering the inherent resistance profiles of the bacterial species involved. The specific antibiotics applied are detailed in Table 4. Different antibiotics were used for each bacterial species. Specific antibiotics for *S. aureus* included FOS, RIF, CHL, CMN, ERY, FTN, KMN, GMN, FOX, AMP, PNG, FAD, NXN, NIR, CIP, MXF, and RIF, while those for *Salmonella spp.* and *E. coli* included FOX, CRO, TCC, FIX, FEP, MEC, AMP, TIC, IPM, CXM, NAL, CIP, NXN, NIR, AKN, TGC, TMP, FOS, and NTM.

Plates were incubated within 15 minutes of disc placement, and the zones of inhibition around the antibiotic discs were measured using an automated system (ADAGIO). The diameters of these inhibition zones were used to determine the sensitivity of the isolates, classified as sensitive (S), resistant (R), or intermediate (I), according to the specific criteria for each bacterium [16].

#### 2.4.2. Detection of Genes Encoding Antibiotic Resistance

This analysis included multiplex PCR amplification of beta-lactam resistance genes (*blaTEM*, *blaSHV, and blaCTX-M*) and fluoroquinolone resistance genes (*qnrA*, *B,* and *S*) in *E. coli* and *Salmonella* species. The method used is that described by the authors in [41]. Indeed, PCR reactions were performed using 2 *μ*L of DNA template (density of 10 ng/*μ*L), 4 *μ*L of Master Mix (5X), 1 *μ*L of each primer (a total of 6 *μ*L per multiplex), and 8 *μ*L of H2O. The reaction mix has a final volume of 20 *μ*L. In addition, the *mecA* gene, responsible for meticillin resistance, was amplified in staphylococci. The primers for these resistance genes can be found in Table 5. The reaction mixture used for this procedure was identical to that employed in the virulence gene testing. Specific PCR conditions tailored to these resistance genes are detailed in Table 6.

## 3. Results and Discussion

### 3.1. Results

#### 3.1.1. Prevalences of Virulence Genes in Bacterial Isolates Isolated from 4th-Range Salads

The study identified the presence of key virulence genes within isolates of *S. aureus* and *E. coli* isolated from ready-to-eat salads. For *S. aureus,* the sea gene showed a prevalence of 55.55%, detected in five samples (SA, SCOM, SN, SChV, and SME). The sec gene was less prevalent, found in only one sample (SCOM) at 11.11%. The sed gene was identified in four samples (Rq, SA, SCA, and SCOM), with a prevalence of 44.44%.

**Figure 1:**
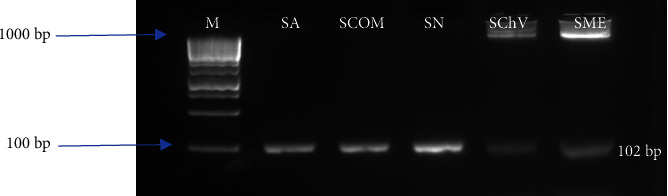
Electrophoretic profile of the amplification product of the *S. aureus* virulence gene (*sea*). M: molecular marker (100 bp); isolates from SA: Aperitif lettuces, SChV: green oak leaf salads, SN: Nicoise salads, SCOM: mixed salads, and SME: Meli melo salads.

**Figure 2:**
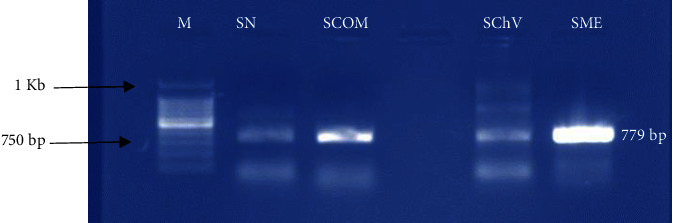
Electrophoretic profile of *E. coli* virulence gene amplification product (*stx2*). M: molecular marker (250 bp); isolates from SN: Nicoise salads, SCOM: mixed salads, SME: Meli melo salads, and SChV: green oak leaf salads.

In *E. coli*, the virulence genes detected included *ST* and *stx2*, with prevalences of 62.50% (five samples: SN, SCOM, SChV, SFR, and SME) and 50% (four samples: SN, SCOM, SChV, and SME), respectively. Detailed data on these findings are available in Table 7. In addition, electrophoretic profiles illustrating the amplification products of the *sea* and *stx2* genes are presented in Figures 1 and 2, respectively.

#### 3.1.2. Antibiotic Resistance Profile of Isolated Isolates

In this study, the antibiotic resistance profiles of bacterial isolates isolated from ready-to-eat salads were thoroughly investigated. *E. coli* exhibited a high level of resistance, with all isolates (100%) resistant to cefuroxime, and a similarly high resistance was observed for cefoxitin and ampicillin at 87.5%. A lower resistance rate was noted for imipenem at 12.5%, while ciprofloxacin displayed a moderate resistance level of 37.5% (Table 8). For *Salmonella spp.*, resistance to beta-lactam antibiotics was significant, with 42.85% of isolates resistant to cefoxitin and 57.15% to ampicillin*. S. aureus* isolates demonstrated extensive resistance across various antibiotics, with a complete resistance (100%) to benzylpenicillin and substantial resistance rates of 66.66% for both fosfomycin and erythromycin. In addition, resistance was noted at 55.55% for ampicillin, kanamycin, gentamicin, norfloxacin, and rifampicin, while 44.44% of isolates were resistant to cefoxitin and moxifloxacin (Table 8). These findings underscore the critical challenge of combating antibiotic resistance in foodborne pathogens and highlight the need for stringent food safety regulations and proactive antibiotic stewardship.

#### 3.1.3. Prevalence of Antibiotic Resistance Genes and Phenotypes in Isolated Isolates

A comprehensive analysis of resistance genes in *E. coli*, *Salmonella*, and *S. aureus* isolated from ready-to-eat salads was conducted. Thus, the significant resistance of the *E. coli* species to ticarcillin + clavulanic acid (TCC) and cephalosporin (cefoxitin, cefixime, and cefuroxime) results in the *ESBL* phenotype (50% of isolates). However, 10.52% of beta-lactam resistant species tested positive for the *blaTEM* resistance gene. Fluoroquinolone resistance (FQ-R) was identified through the presence of *qnrA* (2.26%), *qnrB* (5.26%), and *qnrS* (5.26%). The presence of resistance genes in *E. coli* isolates reveals high resistance to fluoroquinolones (25%). No resistance genes were detected in *Salmonella* isolates. However, most *S. aureus* isolates (55.55%, or 5 out of 9) harbored the *mecA* gene, indicative of the Meti-R *phenotype*, reflecting meticillin resistance. In addition, resistance to aminoglycosides was observed in 55.55% of *S. aureus* isolates for both gentamicin and kanamycin, contributing to a KTG phenotype affecting 44.44% of these isolates. The MLSB phenotype, linked to resistance to macrolide-lincosamide-streptogramin B antibiotics such as clindamycin (55.55%) and erythromycin (66.66%), was also prevalent in 44.44% of the *S. aureus* isolates.

**Figure 3:**
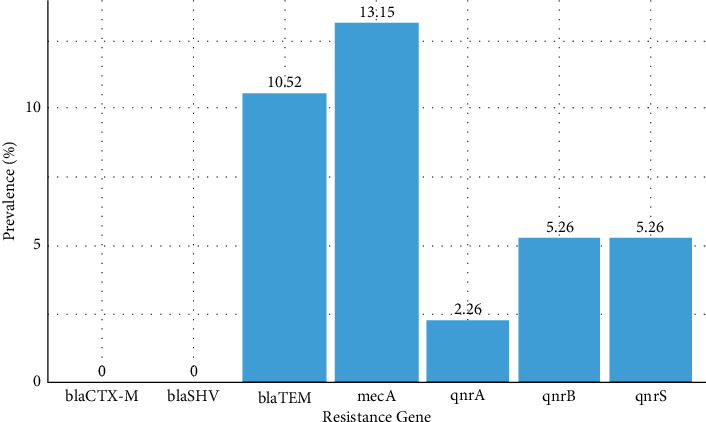
Prevalence of antibiotic resistance genes.

**Figure 4:**
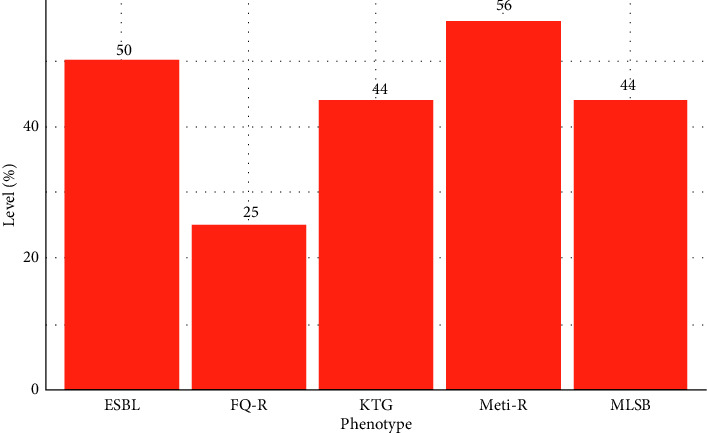
Detected antibiotic resistance phenotypes.

These findings on the prevalence of antibiotic resistance genes and corresponding phenotypes are depicted in Figure 3 for genes and Figure 4 for phenotypes.

### 3.2. Discussion

The analysis of virulence and antibiotic resistance in bacterial isolates isolated from ready-to-eat salads sold in supermarkets in Abidjan revealed notable findings. In *E. coli* isolates, the presence of *stx2* genes was detected in four samples, demonstrating a prevalence of 50%. In addition, the *ST* gene was found in five samples, corresponding to a 62.50% prevalence. Notably, neither *stx1* nor *LT* genes were identified in any of the samples.

Among the four virulence genes (*stx1, stx2, ST,* and *LT*) studied, only *stx2* was linked to Shiga toxin *(STEC*) production. A study in the United States found similar trends where the *stx1* gene was absent in tested isolates, whereas 93.1% carried the *stx2* gene [23]. Moreover, an investigation in Iran revealed a high prevalence of *stx2* genes in cattle feces, supporting the potential for fecal contamination in agricultural settings [13].

Regarding the toxins associated with thermolabile (*LT*) and thermostable (*ST*) toxins in *E. coli*, only the *ST* gene was found, detected in five samples representing a prevalence of 62.50%. Comparable research conducted in Casablanca identified the *ST* gene in certain food products, suggesting possible fecal contamination through irrigation water used in market gardening [24].

Analysis of *S. aureus* in this study revealed the presence of the *sea, sec,* and *sed* genes with prevalences of 55.55%, 11.11%, and 44.44%, respectively. The results of another study showed the absence of enterotoxin genes in ready-to-eat salads, suggesting significantly better hygiene conditions in the production of 4th-range salads [25] than this. In addition, the literature suggests that the presence of certain preservatives such as lactic acid, produced by bacteria, can inhibit *SE* formation in foods, potentially via an extracellular protease, leading to a decrease in enterotoxin levels under specific conditions [29]. In this context, the detection of enterotoxin A, C, and D genes in *S. aureus* in the salads analyzed could indicate deficiencies in personnel hygiene, potentially involving healthy carriers of these genes.

The phenotypic characterization performed on food pathogens isolated from ready-to-eat salads sold in hypermarkets in Abidjan highlights significant concerns, particularly the presence of multiresistant bacteria. These pathogens, which survive without heat treatment in 4th-range foods, pose severe health risks, potentially leading to fatal outcomes for consumers. During this study, antibiotic susceptibility testing revealed a pronounced resistance in *E. coli* to beta-lactam antibiotics, with 100% resistance to cefuroxime (CXM) and 87.5% resistance to both ampicillin (AMP) and cefoxitin (FOX). Similarly, *Salmonella* spp. showed resistance rates of 42.85% to cefoxitin (FOX) and 57.14% to ampicillin (AMP). These findings align with those from other regions; for example, a study in northern California found 76% of *E. coli* isolates resistant to ampicillin and lower resistance to cefoxitin (23%) and cefuroxime (20%) [26]. Contrastingly, a study in southwestern Nigeria reported a 65.7% resistance rate of *E. coli* to cefuroxime [27], while a different study in Abidjan found no resistance in *E. coli* to cefuroxime [28], suggesting variability in bacterial resistance based on the source of salad ingredients. Moreover, resistance to fluoroquinolones was also noted, closely mirroring results from other studies on ready-to-eat foods, with 61.29% resistance to ciprofloxacin observed [29]. Similar research found resistance rates to ciprofloxacin at 8.3% and to carbapenems at 5% [30]. However, this study found a low resistance in *E. coli* to imipenem at 2.9%, akin to findings by another study [31].

For *Salmonella spp.,* the observed resistance profile in this study aligns with the significant resistance to ampicillin reported elsewhere, such as 88% in *S. enteritidis* [32] and 89.9% in Nigerian samples [33]. Additional work on retail meat revealed a notable resistance in *Salmonella* serotypes to ampicillin at 29% and to cefoxitin at 30.43%, further supporting the patterns of resistance found in this study [34]. Antibiotic susceptibility testing of *S. aureus* isolates revealed significant resistance levels to various antibiotic families. High resistance rates were observed with the beta-lactam antibiotics, notably cephalosporins such as cefoxitin (44.44%) and penicillins, with a 100% resistance to benzylpenicillin and 55.55% to ampicillin. For aminoglycosides, resistance levels were 55.55% for both kanamycin (KMN) and gentamicin (GMN). Resistance to macrolides was also substantial, with erythromycin (ERY) at 66.66% and clindamycin (CMN) at 55.55%. Resistance to fluoroquinolones varied, peaking at 55.55% for norfloxacin (NXN). Resistance to beta-lactam antibiotics in *S. aureus* can be attributed to the production of beta-lactamase, which hydrolyzes the beta-lactam ring of penicillins or intrinsic resistance mechanisms such as modification of penicillin-binding proteins (PBPs) or acquisition of new PBPs. This phenomenon of meticillin resistance, which leads to broad-spectrum resistance to all beta-lactam antibiotics, is described in references [35, 36]. Macrolide resistance generally involves the action of a methylase enzyme that modifies the 23S subunit of ribosomal RNA. Comparatively, these resistance patterns correspond closely to results obtained in Côte d'Ivoire, where one study documented a 50% resistance rate to erythromycin [37].

Fluoroquinolone resistance often involves three primary mechanisms: target modification through mutations in the *grlA* or *grlB* genes of topoisomerase IV [38]. Other significant resistance observed included fosfomycin (66.66%), rifampicin (50%), and chloramphenicol (37%). A study conducted in Greece by the authors in [39] explored the prevalence, distribution, and antibiotic susceptibility of *S. aureus* in ready-to-eat salads and environmental and personnel samples from a salad production facility. They found *S. aureus* in 27% of samples, with most isolates showing resistance to 2–5 antibiotics, including fosfomycin. This aligns with the findings in the current study, which showed higher resistance rates to fosfomycin (82.6%), rifampicin (55.55%), and chloramphenicol (44.44%), suggesting a more pronounced resistance profile compared to the Greek study.

During this study, the assessment of antibiotic resistance profiles necessitated identifying specific resistance genes in *E. coli*, *Salmonella,* and *S. aureus* isolates from ready-to-eat salads. *E. coli* resistance to beta-lactam antibiotics in this study was high, with a 50% ESBL (extended-spectrum beta-lactamase) phenotype. However, the *blaTEM* gene was only identified in 10.52% of isolates. In addition, fluoroquinolone resistance was evident via the *qnrA* gene (2.26%), In addition, resistance to fluoroquinolones was evident through the *qnrA* (2.26%), *qnrB* (5.26%), and *qnrS* (5.26%) genes, which collectively underpinned fluoroquinolone resistance in 25% of cases. These findings align with those reported in [40]. In *S. aureus*, the *mecA* gene was detected with a prevalence of 13.15%, accounting for 55.55% of the isolates and indicating resistance to meticillin, characteristic of the Meti-R phenotype. The study also found significant resistance to aminoglycosides, specifically gentamycin and kanamycin, each at 55.55%, correlating with the KTG phenotype in 44.44% of the samples. In addition, high rates of resistance to clindamycin (55.55%) and erythromycin (66.66%) were observed, contributing to the MLSB phenotype in 44.44% of the isolates. A study cited as [39] noted that in MRSA isolates, resistance rates exceed 90%, mirroring the findings of this study where all identified multiresistant isolates exhibited four resistance phenotypes: Meti-R, KTG, MLSB, and resistance to fluoroquinolones. Such elevated resistance levels could significantly contribute to the rise in nosocomial infections and complicate adherence to antibiotic therapy protocols by both patients and healthcare providers. Ultimately, this situation underscores the critical need for standardized antibiogram practices to guide effective antibiotic treatments, especially in cases where standard protocols may not be adequately followed.

## 4. Conclusion

The study of virulence and antibiotic resistance in bacterial isolates isolated from ready-to-eat salads has underscored significant health risks to consumers. Particularly, the pathogenic potential of *E. coli* was demonstrated through the identification of *stx2* and *ST* genes with prevalences of 50% and 62.50%, respectively. Similarly, the detection of *sea, sec,* and *sed* genes in *S. aureus* isolates, having prevalences of 55.55%, 11.11%, and 44.44%, respectively, highlights the virulent nature of these pathogens. The antibiotic resistance profiles further reveal that isolates of *S. aureus* and *E. coli* exhibit multiple resistances to antibiotics, complicating treatment options. Notably, *E. coli* isolates showed substantial multiresistance, evidenced by the presence of *blaTEM* genes (10.52% prevalence), which contribute to an ESBL phenotype in 50% of the isolates. In addition, the detection of *qnrA* (2.26%), *qnrB* (5.26%), and *qnrS* (5.26%) genes indicates a 25% prevalence of resistance to quinolones. *S. aureus* isolates displayed resistance to meticillin, as marked by the *mecA* gene (13.15% prevalence) and a Meti-R rate of 55.55%. High resistance rates were also observed in aminosides and macrolides, characterized by KTG and MLSB phenotypes, both at 44.44%. These findings suggest that the consumption of contaminated ready-to-eat salads could lead to therapeutic challenges in treating human infections due to the high prevalence of antibiotic-resistant bacteria. As these salads become a more common part of the diet, it is imperative to enforce stringent safety measures and monitoring to mitigate the risk of consumer poisoning and limit the spread of multiresistant bacteria. This approach is crucial in preserving the efficacy of antibiotics and ensuring the health and safety of consumers.

## Figures and Tables

**Table 1:** Detection of pathogenic bacteria in ready-to-eat salads.

Salads	Germs		
	*E. coli*	*S. aureus*	*Salmonella spp*.
100% curly heart (Cf)	−	−	−
Baby spinach (Ep)	−	−	−
Young shoots (JP)	−	−	+
Lamb's lettuce (Ma)	−	−	−
Lamb's lettuce + rocket (MR)	−	−	**+**
Rocket (Rq)	−	**+**	−
Aperitif salads (SA)	−	**+**	**+**
Salads carrots (SCA)	**+**	**+**	−
Salads cabbage (SCH)	**+**	−	**+**
Green oak leaf salads (SChV)	**+**	**+**	**+**
Mixed salads (SCOM)	**+**	**+**	−
Pineapple fruit salads (SFA)	−	−	−
Mixed fruit salads (SFC)	**+**	−	−
Pineapple + mango fruit salads (SFAM)	−	−	−
Papaya + pineapple fruit salads (SFPA)	−	**+**	−
Papaya + lemon fruit salads (SFPC)	−	−	−
Grapefruit salads (SFR)	+	**+**	**+**
Meli melo salads (SME)	+	**+**	−
Nicoise salads (SN)	**+**	**+**	**+**

**+**, Presence of pathogens; −, pathogen-free.

**Table 2:** Primers used for the detection of virulence genes in *E. coli* and *S. aureus*.

Germs	Genes	Sequences (5′->3′)	Sizes (bp)	References
*E. coli*	*stx1*	F: ACACTGGATGATCTCAGTGG R: CGTAATCCCCCTCCATTATG	614	[17]
	*stx2*	F: CCATGACAACGGACAGCAGTT R: CCTGTCAACTGAGCAGCACTTTG	779	[17]
	*LT*	F: GCGACAAATTATACCGTGCT R: CCGAATTCTGTTATATATGT	708	[18]
	*ST*	F: CTGTATTTGTCTTTTTCACCT R: GCACCCGGTACAAGCAGGAT	182	[18]

*S. aureus*	*sea*	F: GGTTATCAATGTGCGGGTGG R: CGGCACTTTTTTCTCTTCGG	102	[15]
	*seb*	F: GTATGGTGGTGTAACTGAGC R: CCAAATAGTGACGAGTTAGG	164	[15]
	*sec*	F: AGATGAAGTAGTTGATGTGTATGG R: CACACTTTTAGAATCAACCG	451	[15]
	*sed*	F: CCAATAATAGGAGAAAATAAAAG R: ATTGGTATTTTTTTTCGTTC	278	[15]
	*see*	F: AGGTTTTTTCACAGGTCATCC R: CTTTTTTTTCTTCGGTCAATC	209	[15]

*stx1*, Shiga toxin 1; *stx2*, Shiga toxin 2; *LT*, heat-labile toxin; *ST*, heat-stable toxin; *sea, seb, sec, sed,* and *see*: *Staphylococcal enterotoxins*, a–e.

**Table 3:** Virulence gene amplification program.

Amplification steps	Amplification conditions (temperature and weather)		
	*stx1*/*stx2*	*ST*/*LT*	*sea*/*seb*/*sec*/*sec*/*sed*/*see*
Initial denaturation	94°C/5 min	94°C/5 min	94°C/5 min
Cyclic denaturation	94°C/30 s	94°C/45 s	94°C/2 min
Hybridization	56°C/30 s	60°C/1 min	57°C/2 min
Cyclic elongation	72°C/30 s	72°C/1 min	72°C/1 min
Final elongation	72°C/10 min	72°C/7 min	72°C/7 min
Number of cycles	30	35	35

**Table 4:** List of antibiotic discs tested on bacterial isolates.

Antibiotic families	Antibiotics	Code	Fillers (*μ*g)
Beta-lactam antibiotics	Benzylpenicillin	PENG	1 IU
	Cefoxitin	FOX	30
	Ceftriaxone	CRO	30
	Ticarcillin + clavulanic acid	TCC	85
	Cefixime	FIX	5
	Cefepime	FEP	30
	Mecillinam	MEC	10
	Ticarcillin	TIC	75
	Cefuroxime	CXM	30
	Ampicillin	AMP	10

Macrolide, lincosamide, and streptogramin	Clindamycin	CMN	2
	Erythromycin	ERY	15

Fluoroquinolones	Norfloxacin	NXN	10
	Nitroxoline	NIR	30
	Ciprofloxacin	CIP	5
	Moxifloxacin	MXF	5
	Nalidixic acid	NAL	30

Carbapenem	Imipenem	IPM	10
	Meropenem	MEN	10

Tetracycline	Tigecycline	TGC	15

Aminosides	Kanamycin	KMN	30
	Gentamicin	GMN	10
	Netilmicin	NTM	10
	Amikacin	AKN	30

Others	Fusidic acid	FAD	10
	Rifampicin	RIF	5
	Fosfomycin	FOS	200
	Chloramphenicol	CHL	30
	Nitrofuran	FTN	300

**Table 5:** Primers used for the detection of resistance genes in *E. coli, Salmonella spp.*, and *S. aureus*.

Germs	Genes	Sequences (5′->3′)	Sizes (bp)	References
*E. coli* and *Salmonella spp.*	*bla*_*TEM*_	F: ATGAGTATTCAACATTTCCGTG R: TTACCAATGCTTAATCAGTGAG	840	[19]
	*bla*_*SHV*_	F: TTTATGGCGTTACCTTTGACC R: ATTTGTCGCTTCTTTACTCGC	1051	[20]
	*bla*_*CTX* –*M*_	F: GGTTAAAAAATCACTGCGTC R: TTGGTGACGATTTTAGCCGC	863	[21]
	*qnrA*	F: GATAAAGTTTTTCAGCAAGAGG R: ATCCAGATCGGCAAAGGTTA	543	[21]
	*qnrB*	F: GACAGAAACAGGTTCACCGGT R: CAAGACGTTCCAGGAGCAACG	469	[21]
	*qnrS*	F: ACGACATTCGTCAACTGCAA R: TAAATTGGCAACCTGTAGGC	417	[21]

*S. aureus*	*mecA*	F: TGCTATCCACCCTCAAACAGG R: AACGTTGTAACCACCCCAAGA	286	[22]

*blaTEM, blaSHV, and blaCTX-M*: beta-lactam resistance genes; *qnrA, qnrB, and qnrS*: fluoroquinolone resistance marker genes; *mecA*: meticillin resistance; *E. coli*, *Escherichia coli; S. aureus, Staphylococcus aureus.*

**Table 6:** Resistance gene amplification conditions.

Amplification steps	Temperature condition/times		
	blaCTX-M, blaTEM, blaSHV	*qnrA, qnrB, qnrS*	*mecA*
Initial denaturation	94°C/5 min	95°C/5 min	94°C/4 min
Cyclic denaturation	94°C/1 min	95°C/30 s	94°C/30 s
Hybridization	60°C/1 min	60°C/30 s	60°C/1 min
Cyclic elongation	72°C/1 min	72°C/1 min	72°C/2 min
Final elongation	72°C/7 min	72°C/10 min	72°C/4 min
Number of cycles	(30)	(30)	(35)

**Table 7:** Prevalence of virulence genes in *E. coli* and *S. aureus*.

Isolates	Genes sought	Number of samples	Prevalences (%)
*E. coli*	*LT*	8	0 (0)
	*ST*	8	5 (62.50)
	*stx1*	8	0 (0)
	*stx2*	8	4 (50)

*S. aureus*	*sea*	9	5 (55.55)
	*seb*	9	0 (0)
	*sec*	9	1 (11.11)
	*sed*	9	4 (44.44)
	*see*	9	0 (0)

*stx1*, Shiga toxin 1; *stx2*, Shiga toxin 2; *LT*, heat-labile toxin; *ST*, heat-stable toxin; *sea, seb, sec, sed,* and *see*: *Staphylococcal enterotoxins,* a–e.

**Table 8:** Resistance profile of bacterial isolates isolated from ready-to-eat salads.

Antibiotic families	Molecular	*E. coli*	*Salmonella spp.*	*S. aureus*
Beta-lactam antibiotics	Benzylpenicillin (PENG).1 IU	0/8 (0)	0/7 (0)	9/(100)
	Cefoxitin (FOX).30 *μ*g	7/8 (87.5)	3/7 (42.85)	4/9 (44.44)
	Ceftriaxone (CRO).30 *μ*g	0/8 (0)	0/7 (0)	0/9 (0)
	Ticarcillin + clavulanic acid (TCC).85 *μ*g	4/8 (50)	0/7 (0)	0/9 (0)
	Cefixime (FIX).5 *μ*g	3/8 (37.5)	0/7 (0)	0/9 (0)
	Cefepime (FEP).30 *μ*g	0/8 (0)	0/7 (0)	0/9 (0)
	Mecillinam (MEC).10 *μ*g	2/8 (25)	0/7 (0)	0/9 (0)
	Ticarcillin (TIC).75 *μ*g	4/8 (50)	0/7 (0)	0/9 (0)
	Cefuroxime (CXM).30 *μ*g	8/8 (100)	0/7 (0)	0/9 (0)
	Ampicillin (AMP).10 *μ*g	7/8 (87.5)	4/7 (57.14)	5/9 (55.55)

Macrolide, lincosamide, and streptogramin	Clindamycin (CMN).2 *μ*g	0/8 (0)	0/7 (0)	5/9 (55.55)
	Erythromycin (ERY).15 *μ*g	0/8 (0)	0/7 (0)	6/9 (66.66)

Fluoroquinolones	Norfloxacin (NXN).10 *μ*g	2/8 (25)	0/7 (0)	5/9 (55.55)
	Nitroxoline (NIR).30 *μ*g	3/8 (37.5)	0/7 (0)	0/9 (0)
	Ciprofloxacin (CIP).5 *μ*g	3/8 (37.5)	0/7 (0)	3/9 (33.33)
	Moxifloxacin (MXF).5 *μ*g	0/8 (0)	0/7 (0)	4/9 (44.44)
	Nalidixic acid (NAL).30 *μ*g	1/8 (12.5)	0/7 (0)	0/9 (0)

Carbapenem	Imipenem (IPM).10 *μ*g	1/8 (12.5)	0/7 (0)	0/9 (0)
	Meropenem (MEN).10 *μ*g	0/8 (0)	0/7 (0)	0/9 (0)

Tetracycline	Tigecycline (TGC).15 *μ*g	0/8 (0)	0/7 (0)	0/9 (0)

Aminosides	Kanamycin (KMN).30 *μ*g	0/8 (0)	0/7 (0)	5/9 (55.55)
	Gentamicin (GMN).10 *μ*g	0/8 (0)	0/7 (0)	5/9 (55.55)
	Netilmicin (NTM).10 *μ*g	2/8 (25)	0/7 (0)	0/9 (0)
	Amikacin (AKN).30 *μ*g	2/8 (25)	0/7 (0)	0/9 (0)

Others	Fusidic acid (FAD).10 *μ*g	0/8 (0)	0/7 (0)	6/9 (66.66)
	Rifampicin (RIF).5 *μ*g	0/8 (0)	0/7 (0)	5/9 (55.55)
	Fosfomycin (FOS).200 *μ*g	0/8 (0)	0/7 (0)	6/9 (66.66)
	Chloramphenicol (CHL).30 *μ*g	0/8 (0)	0/7 (0)	4/9 (44.44)
	Nitrofuran (FTN).300 *μ*g	0/8 (0)	0/7 (0)	3/9 (33.33)

## Data Availability

The data used to support the findings of this study are included within the article.
